# Combined Hesperidin and Gemcitabine Therapy Modulates Apoptosis and Angiogenesis Pathways in ISHIKAWA Human Endometrial Adenocarcinoma Cells

**DOI:** 10.3390/medicina61091599

**Published:** 2025-09-04

**Authors:** Yasemin Afşin, İlhan Özdemir, Veysel Toprak, Mehmet Cudi Tuncer, Şamil Öztürk

**Affiliations:** 1 Department of Gynecology and Obstetrics, Private Batman Life Hospital, Batman 72040, Turkey; dryaseminafsin@outlook.com; 2 Department of Histology Embryology, Faculty of Medicine, Kahramanmaraş Sütçü İmam University, Kahramanmaraş 46100, Turkey; ilhanozdemir25@yandex.com; 3 Department of Gynecology and Obstetrics, Faculty of Medicine, Private Metrolife Hospital, Şanlıurfa 63320, Turkey; drveysel21@outlook.com; 4 Department of Anatomy, Faculty of Medicine, Dicle University, Diyarbakır 21280, Turkey; 5 Vocational School of Health Services, Çanakkale Onsekiz Mart University, Çanakkale 17100, Turkey; ozturksamil@outlook.com

**Keywords:** endometrial cancer, apoptosis, MTT, hesperidin, gemcitabine, angiogenesis

## Abstract

*Background and Objectives:* Endometrial adenocarcinoma is among the most prevalent malignancies of the female reproductive system, and therapeutic options remain limited, particularly in advanced stages. In recent years, natural agents, especially flavonoids, have gained considerable interest for their capacity to enhance the effectiveness of chemotherapeutic drugs and modulate tumor-related molecular mechanisms. Hesperidin, a citrus-derived flavonoid, is recognized for its antioxidant and anti-inflammatory effects, while Gemcitabine, a nucleoside analog, is widely used in cancer treatment. Investigating their combined effects on endometrial carcinoma cells could yield novel insights into multimodal therapeutic development. This current study aimed to assess the impact of Hesperidin (Hes) and Gemcitabine (Gem) on ISHIKAWA cells, a human endometrial adenocarcinoma model, with particular attention to pathways associated with hypoxia, angiogenesis, apoptosis, and oxidative stress. *Materials and Methods:* ISHIKAWA cells were treated with varying concentrations of Hes (50–200 µM) and Gem (10–50 nM), either individually or together, for 24 and 48 h. Cell viability was determined using the MTT assay, while apoptosis was measured by Caspase-3/7 activity and NucBlue nuclear staining. Intracellular reactive oxygen species (ROS) generation was quantified via DCFH-DA fluorescence. Expression levels of HIF-1α, VEGF, Bax, Bcl-2, and Caspase-3 were examined by *RT-qPCR*. Synergistic interactions were analyzed with the Chou–Talalay combination index. Biological enrichment was further explored using Gene Ontology (GO) and Kyoto Encyclopedia of Genes and Genomes (KEGG) analyses. *Results*: Both Hes and Gem significantly decreased ISHIKAWA cell viability in a concentration- and time-dependent manner (*p* < 0.001). The combined treatment induced stronger apoptotic effects, as reflected by increased Caspase-3/7 activity and nuclear morphological changes. *RT-qPCR* demonstrated upregulation of Bax and Caspase-3, together with downregulation of Bcl-2, HIF-1α, and VEGF. While Hes reduced intracellular ROS, Gem elevated it; their combination produced a balanced oxidative response. All dose combinations displayed strong synergism (CI < 1). GO and KEGG enrichment confirmed the involvement of apoptosis-, angiogenesis-, and hypoxia-related pathways. *Conclusions*: Co-treatment with Hes and Gem exhibits synergistic anticancer activity in endometrial cancer cells by promoting apoptosis, suppressing angiogenesis- and hypoxia-related gene expression, and modulating oxidative stress. This combined therapeutic approach highlights its potential as a promising adjuvant option, warranting further evaluation in in vivo and translational studies.

## 1. Introduction

Endometrial cancer represents one of the most prevalent malignancies of the female reproductive system, with its incidence steadily rising, particularly in developed countries [1]. Arising from the epithelial lining of the endometrium, the disease is typically treated with surgery and adjuvant therapies when detected at an early stage. In contrast, advanced or recurrent forms present limited therapeutic alternatives, often leading to unfavorable clinical outcomes [2]. Therefore, innovative therapeutic approaches that move beyond conventional strategies are urgently required to address resistance mechanisms and disease progression.

Within the tumor microenvironment, hypoxia, a condition of reduced oxygen availability, plays a pivotal role in driving therapeutic resistance. Under hypoxic conditions, tumor cells activate adaptive mechanisms that promote their survival, including the induction of angiogenesis, the inhibition of apoptosis, and the facilitation of metastasis [3]. Central to this adaptation is *HIF-1α*, a transcription factor regulating the expression of genes linked to angiogenesis and metabolic reprogramming [4]. Hypoxia markedly increases *VEGF* expression, a crucial angiogenic mediator that fosters new vessel formation and ensures a continuous nutrient and oxygen supply for proliferating tumor cells. Additionally, hypoxia-mediated signaling attenuates apoptotic pathways, further reinforcing chemoresistance [5].

Considering these interconnections, targeting both hypoxia- and angiogenesis-related pathways alongside stimulation of apoptosis has been proposed as a potential therapeutic avenue in endometrial cancer. Hes, a flavanone glycoside derived from citrus fruits, is recognized for its antioxidant, anti inflammatory, and anticancer properties. Previous studies have shown that Hes can suppress tumor growth, block angiogenesis, and trigger apoptosis in different cancer models [6]. In endometrial carcinoma cells, Hes has specifically been found to initiate programmed cell death by modulating estrogen receptor-independent signaling and shifting the *Bax/Bcl-2* ratio toward apoptosis [7,8]. Nevertheless, its exact role in regulating hypoxia associated pathways and its potential synergism with standard chemotherapeutics remain incompletely defined.

Gem, a nucleoside analog acting as a DNA synthesis inhibitor, is widely used for the management of solid tumors. However, its clinical benefit is frequently limited by acquired resistance, low bioavailability, and systemic toxicity [9]. To address these drawbacks, natural bioactive compounds have been explored as adjuvants to enhance Gem effectiveness [10]. Flavonoid-based combination regimens may not only augment anticancer activity but also mitigate chemotherapy induced toxicity owing to their antioxidant characteristics.

Based on this rationale, the present study was conducted to investigate the molecular crosstalk and potential synergistic interactions between Hes and Gem in ISHIKAWA human endometrial adenocarcinoma cells. The analysis focused on critical pathways associated with hypoxia (*HIF-1α*), angiogenesis (*VEGF*), and apoptosis (*Bax*, *Bcl-2*, *Caspase-3*). Furthermore, we aimed to determine whether combining a natural flavonoid with a conventional chemotherapeutic could amplify apoptotic signaling while concurrently suppressing tumor promoting mechanisms. By evaluating this dual targeting approach, the study intends to contribute to the development of innovative adjuvant strategies against chemoresistance and to improve treatment outcomes in endometrial cancer.

## 2. Material and Methods

### 2.1. Maintenance and Propagation of Human Endometrial Adenocarcinoma (ISHIKAWA) Cells

For this study, the ISHIKAWA human endometrial adenocarcinoma cell line was employed as the in vitro model. The line was procured from the American Type Culture Collection (ATCC^®^ HTB-112™, Manassas, VA, USA), with authentication information available in the Cellosaurus database (CVCL_2529). Cells were grown in Dulbecco’s Modified Eagle Medium (DMEM; Gibco, Thermo Fisher Scientific, Waltham, MA, USA) supplemented with 10% fetal bovine serum (FBS; Gibco), 1% penicillin streptomycin (100 U/mL penicillin, 100 µg/mL streptomycin), and 2 mM L-glutamine. Cultures were maintained in a humidified environment at 37 °C with 5% CO_2_.

Cell morphology and density were routinely examined using an inverted phase contrast microscope. Once cultures reached 80–90% confluence, cells were detached with 0.25% trypsin EDTA (Gibco) and reseeded at a 1:3 split ratio into fresh flasks. To ensure experimental reproducibility and maintain cellular characteristics, all assays were conducted using passages 4 to 10.

### 2.2. Preparation and Application of Hesperidin and Gemcitabine Treatments

Stock solutions of Hes and Gem (Sigma-Aldrich, St. Louis, MO, USA) were prepared in dimethyl sulfoxide (DMSO; Sigma-Aldrich) at final concentrations of 100 mM and 10 mM, respectively. After dissolution, the solutions were vortexed and sterilized using 0.22 µm syringe filters. Working dilutions were freshly prepared in complete culture medium immediately prior to each experiment.

For dose response assays, Hes was tested at 0, 10, 25, 50, and 100 µM, while Gem was applied at 0, 1, 2.5, 5, and 10 µM. In combination experiments, both agents were co-administered at fixed ratios (Hes10 + Gem1, Hes25 + Gem2.5, Hes50 + Gem5, Hes100 + Gem10) to evaluate possible synergistic interactions. Treatments were applied in 100 µL per well in 96-well plates, or scaled proportionally for other culture formats.

To avoid solvent induced cytotoxicity, the maximum DMSO concentration in all experimental groups did not exceed 0.1%. Control groups received medium containing 0.1% DMSO without active agents. Cells were incubated with the treatments for 24 or 48 h at 37 °C in a humidified incubator with 5% CO_2_.

### 2.3. Assessment of Cell Viability via MTT Assay

Cell viability was determined using the MTT colorimetric assay (Sigma-Aldrich, St. Louis, MO, USA), which measures mitochondrial reductase activity as an indicator of metabolically active cells. ISHIKAWA cells were seeded into 96-well flat bottom plates at a density of 5 × 10^3^ cells per well in 100 µL of complete medium and were allowed to attach for 24 h under standard conditions (37 °C, 5% CO_2_, humidified atmosphere).

After exposure to Hes and Gem, or their combinations for 24 or 48 h, 10 µL of MTT solution (5 mg/mL in PBS) was added to each well and incubated for 4 h at 37 °C. Formazan crystals formed in viable cells were dissolved with 100 µL of DMSO per well, followed by 10 min incubation with gentle agitation to ensure complete solubilization.

Absorbance values were measured at 570 nm using a microplate spectrophotometer (Multiskan GO, Thermo Fisher Scientific, Waltham, MA, USA). Wells containing only medium and MTT were used for background subtraction. Cell viability was expressed as a percentage of the untreated control using the formula:

Cell viability (%) = (Absorbance of treated cells/Absorbance of control cells) × 100

All assays were conducted in triplicate and repeated in at least three independent experiments to ensure reproducibility. For graphical representation, a minimum value of 0.001 was substituted in place of zero to enable log scale plotting; this adjustment was applied only for visualization and did not affect statistical analyses or IC_50_ determinations.

### 2.4. Quantification of Angiogenic Activity via VEGF ELISA

The angiogenic activity of Hes, Gem, and their combination was evaluated by measuring VEGF secretion through an enzyme linked immunosorbent assay (ELISA). ISHIKAWA cells were seeded into 24-well plates at a density of 1 × 10^5^ cells per well and cultured for 24 h to allow adherence.

Following treatment for 24 or 48 h, culture supernatants were collected and centrifuged at 1500× *g* for 10 min at 4 °C to remove debris. The clarified samples were stored at −80 °C until further analysis. VEGF levels were quantified using a human VEGF Quantikine ELISA kit (R & D Systems, Minneapolis, MN, USA) according to the manufacturer’s instructions. Standards and samples were added in duplicate to the pre-coated 96-well plate, incubated with assay diluent, and processed through sequential washing, conjugate addition, and substrate reaction. The reaction was stopped with the supplied stop solution, and absorbance was measured at 450 nm with background correction at 570 nm using a microplate reader (Multiskan GO, Thermo Fisher Scientific, Waltham, MA, USA).

Concentrations were interpolated from a standard curve generated with known VEGF values and expressed in pg/mL. Each experimental group was analyzed in triplicate in three independent experiments. The coefficient of determination (R^2^) of the standard curve was consistently ≥0.98, confirming linearity and assay reliability.

### 2.5. Evaluation of Intracellular ROS Levels Using the DCFH-DA Fluorescence Assay

Intracellular ROS levels were examined to determine the antioxidant or pro-oxidant impact of Hes, Gem, and their combination in ISHIKAWA cells. The assay is based on the ROS-dependent conversion of non-fluorescent DCFH DA into the fluorescent compound dichlorofluorescein.

Cells were seeded into black walled, clear bottom 96-well plates at a density of 1 × 10^4^ per well and incubated for 24 h to allow attachment. After 24 or 48 h of treatment, the medium was removed and cells were exposed to 10 µM DCFH DA (diluted in serum free DMEM; Sigma Aldrich, St. Louis, MO, USA) for 30 min at 37 °C in the dark to minimize photooxidation. Wells were then washed twice with phosphate-buffered saline (PBS) to eliminate residual probe.

Fluorescence was recorded with a microplate reader (Multiskan GO, Thermo Fisher Scientific, Waltham, MA, USA) at 485 nm excitation and 535 nm emission. Background fluorescence from wells containing only dye and medium was subtracted. Results were expressed as relative fluorescence units (RFU) normalized against untreated controls.

Hydrogen peroxide (H_2_O_2_, 100 µM, 1 h) served as a positive control to verify assay responsiveness. All conditions were tested in triplicate, and experiments were repeated independently three times.

### 2.6. Quantitative Luminometric Assessment of Caspase-3/7 Enzymatic Activity

The enzymatic activity of *Caspase 3* and *Caspase 7*, which act as central executioners of apoptosis, was determined using the *Caspase Glo 3/7* Assay Kit (Promega, Madison, WI, USA). This method relies on the luminometric detection of substrate cleavage (DEVD aminoluciferin) by activated caspases.

ISHIKAWA cells were plated into white opaque 96-well plates (Corning, New York, NY, USA) at a density of 5 × 10^3^ per well in 100 µL complete medium and incubated for 24 h for attachment. Cells were then treated with Hes (0–100 µM), Gem (0–10 µM), or their combinations for 24 or 48 h under standard culture conditions.

Following treatment, *Caspase Glo 3/7* reagent was equilibrated to room temperature for 30 min, and 100 µL was added to each well already containing 100 µL of culture medium. Plates were mixed gently on an orbital shaker for 30 s to ensure uniformity and then incubated at room temperature for 30 min in the dark to allow substrate cleavage and luminescence generation.

Luminescence was measured on a multimode reader (Glomax Multi Detection System, Promega, Madison, WI, USA) and expressed as relative light units (RLU). Background signal from blank wells (medium plus reagent) was subtracted. Untreated cells were considered as the negative control, whereas treatment with 1 µM staurosporine for 6 h served as the positive control.

Results were normalized to the negative control and reported as fold change in caspase activity. Each condition was tested in triplicate, and experiments were repeated at least three times. Statistical evaluation was carried out using one-way ANOVA followed by Tukey’s post hoc test, with *p* < 0.05 regarded as significant.

### 2.7. Fluorescence-Based Nuclear Morphology Analysis via NucBlue Staining

Nuclear alterations typical of apoptosis, including chromatin condensation and fragmentation, were assessed using NucBlue Live ReadyProbes Reagent (Hoechst 33342 based; Thermo Fisher Scientific, Waltham, MA, USA). This DNA binding dye specifically labels nuclei, allowing the visualization of apoptotic changes by fluorescence microscopy.

ISHIKAWA cells were seeded in sterile black 24-well clear bottom plates at a density of 5 × 10^4^ per well and cultured for 24 h to permit attachment. Following treatment with Hes and Gem, or their combinations for 24 or 48 h, two drops of NucBlue reagent were added directly into each well containing live cells in complete medium, according to the manufacturer’s instructions.

Plates were then incubated for 20 min at 37 °C in the dark to achieve optimal nuclear staining without phototoxicity. Imaging of live cells was carried out using the Thermo EVOS FL Imaging System equipped with a DAPI filter. Both brightfield and fluorescence images were captured at 20× magnification. Apoptotic nuclei were identified by their characteristic morphology, including enhanced fluorescence, condensed chromatin, and fragmented structures.

For quantitative evaluation, apoptotic nuclei were counted in at least five randomly selected microscopic fields per treatment group. The proportion of apoptotic cells was calculated as the percentage of apoptotic nuclei relative to the total cell count.

### 2.8. Quantitative Gene Expression Profiling by RT-qPCR

To evaluate the transcriptional regulation of hypoxia-, angiogenesis-, and apoptosis- related genes, the mRNA expression levels of *HIF-1α*, *VEGF*, *Bax*, *Bcl-2*, and *Caspase-3* were quantified using real time quantitative polymerase chain reaction (*RT-qPCR*).

After 24 or 48 h of drug exposure, total RNA was isolated from ISHIKAWA cells with TRIzol reagent (Thermo Fisher Scientific, Waltham, MA, USA) following the manufacturer’s protocol. RNA concentration and purity were determined with a NanoDrop 2000 spectrophotometer (Thermo Fisher Scientific, Waltham, MA, USA), and only samples with an A260/A280 ratio of 1.8–2.0 were used. RNA integrity was confirmed by 1.5% agarose gel electrophoresis under non-denaturing conditions.

Complementary DNA was synthesized from 1 µg of total RNA using the High Capacity cDNA Reverse Transcription Kit (Thermo Fisher Scientific, Waltham, MA, USA) with the following cycling conditions: 25 °C for 10 min, 37 °C for 120 min, 85 °C for 5 min, and cooling at 4 °C.

RT qPCR reactions were run on a QuantStudio 5 Real Time PCR System (Applied Biosystems, Waltham, MA, USA) with SYBR Green Master Mix (Applied Biosystems) and gene specific primers (Table 1). Each 20 µL reaction contained 10 µL SYBR Green Master Mix, 1 µL forward and reverse primers (10 µM each), 2 µL cDNA, and 6 µL nuclease free water. The amplification protocol consisted of initial denaturation at 95 °C for 10 min, followed by 40 cycles of 95 °C for 15 s and 60 °C for 60 s. Melting curve analysis was performed at the end of each run to verify product specificity. All reactions were conducted in triplicate for each condition.

Relative gene expression was normalized to GAPDH as the internal reference and calculated by the 2^−ΔΔCt^ method. Negative controls without template and without reverse transcription were included in every run to exclude contamination and genomic DNA amplification.

### 2.9. GO and Pathway Enrichment Analysis

GO analysis was performed to determine the functional roles of the differentially expressed genes (*HIF-1α*, *VEGF*, *Bax*, *Bcl-2*, and *Caspase-3*) identified by *RT-qPCR*. These genes were evaluated for enrichment in the major GO categories: biological process (BP), molecular function (MF), and cellular component (CC).

Functional annotation and enrichment were carried out using DAVID version 6.8 (available online: https://david.ncifcrf.gov/ (accessed on 25 June 2025)), with gene symbols mapped against the Homo sapiens reference genome. Significantly enriched GO terms were determined using a modified Fisher’s exact test with Benjamini–Hochberg correction, and terms with an adjusted *p*-value < 0.05 were considered significant.

KEGG pathway enrichment analysis was also conducted to assess the involvement of these genes in canonical pathways. The results highlighted their roles in cellular responses to hypoxia, regulation of angiogenesis, mitochondrial-mediated apoptosis, and stress-activated signaling cascades. The list of genes used in the enrichment analyses is provided in Table 2.

### 2.10. Statistical Analysis

All data were obtained from at least three independent biological replicates, each with technical triplicates. Results are presented as mean ± standard deviation (SD). Data normality was evaluated using the Shapiro–Wilk test, and homogeneity of variances with Levene’s test.

For comparisons among multiple groups, one-way ANOVA was applied. When significant differences were observed, Tukey’s post hoc test was used for pairwise comparisons. Gene expression levels from *RT-qPCR* were analyzed using the 2^−^ΔΔCt method and log-transformed when necessary to satisfy parametric assumptions.

All statistical analyses and graph visualizations were conducted in GraphPad Prism version 9.0 (GraphPad Software, San Diego, CA, USA). Statistical significance was set at *p* < 0.05. Figures and legends denoted levels of significance as *p* < 0.05, *p* < 0.01, and *p* < 0.001. Gene symbols were formatted in italics in accordance with HGNC guidelines, while protein names were written in roman type.

## 3. Results

### 3.1. Effects of Hesperidin, Gemcitabine, and Their Combination on ISHIKAWA Cell Viability

**Figure 1:**
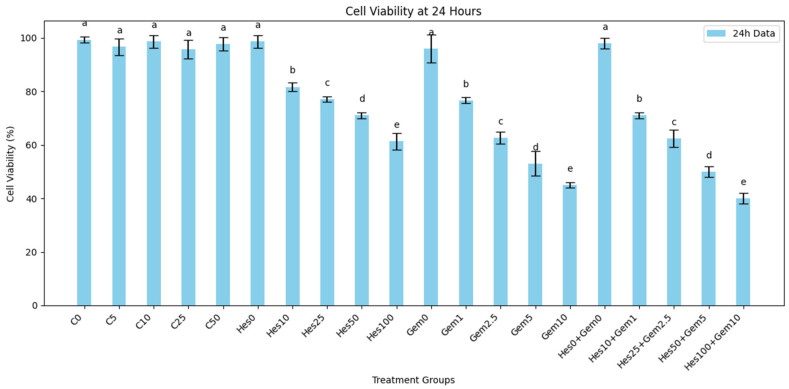
Effects of Hes, Gem, and their combined treatments on ISHIKAWA cell viability after 24 h. Data are expressed as mean ± standard deviation (SD) from three independent assays. Groups sharing the same lowercase letter were not significantly different according to one-way ANOVA with Tukey’s post hoc test (*p* < 0.05). “a” groups (C0, C5, C10, C25, C50, Hes0, Gem0, Hes0 + Gem0) showed no significant change compared with untreated control (C0). “b” groups (Hes10, Gem1, Hes10 + Gem1) exhibited slight but significant decreases (*p* < 0.05), “c” groups (Hes25, Gem2.5, Hes25 + Gem2.5) demonstrated moderate decreases (*p* < 0.01), “d” groups (Hes50, Gem5, Hes50 + Gem5) showed marked decreases (*p* < 0.001), and “e” groups (Hes100, Gem10, Hes100 + Gem10) exhibited the strongest viability reduction (*p* < 0.001 vs. C0).

**Figure 2:**
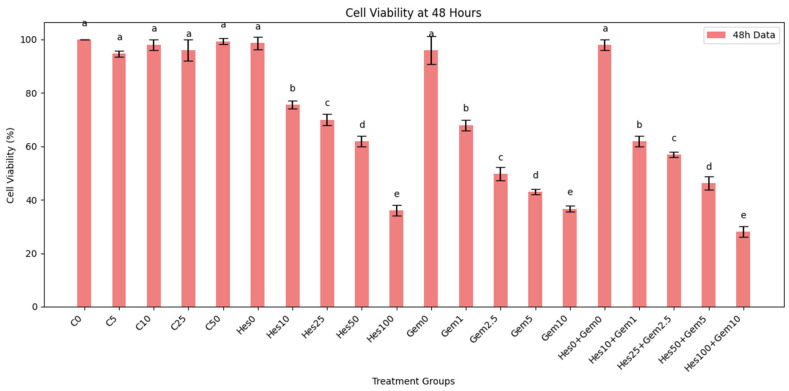
Effects of Hes, Gem, and their combinations on ISHIKAWA cell viability after 48 h. Results are shown as mean ± standard deviation (SD) of three independent replicates. Statistical comparisons were performed using one-way ANOVA with Tukey’s post hoc test (*p* < 0.05). Groups sharing the same lowercase letter did not differ significantly. Category “a” (C0, C5, C10, C25, C50, Hes0, Gem0, Hes0 + Gem0) showed no deviation from untreated control (C0). A stepwise decline in cell viability was observed with increasing concentrations: “b” (Hes10, Gem1, Hes10 + Gem1; *p* < 0.05 vs. C0), “c” (Hes25, Gem2.5, Hes25 + Gem2.5; *p* < 0.01 vs. C0), “d” (Hes50, Gem5, Hes50 + Gem5; *p* < 0.001 vs. C0), and “e” (Hes100, Gem10, Hes100 + Gem10; *p* < 0.001 vs. C0).

Cell viability was evaluated at 24 and 48 h after treatment with Hes, Gem, or their combinations (Figure 1 and Figure 2). Experimental groups included untreated control (C0), vehicle controls (C5−−C50), Hes (Hes10−Hes100), Gem (Gem1−Gem10), and five combination groups (Hes10 + Gem1 to Hes100 + Gem10). Viability was expressed relative to untreated controls.

Control and vehicle groups maintained 90–100% viability at both time points without significant changes. Hes induced a dose- and time-dependent decrease: 80–83% (Hes10), 70–72% (Hes50), and 58–64% (Hes100) at 24 h, with further reduction at 48 h, particularly Hes100 (34–38%, *p* < 0.05 vs. control). Gem monotherapy also reduced viability: 76–78% (Gem1), 49–58% (Gem5), and 44–46% (Gem10) at 24 h; Gem10 dropped to 36–38% at 48 h (*p* < 0.001 vs. control).

Combination therapy enhanced cytotoxicity compared with single agents. Hes10 + Gem1 decreased viability to 70–72% at 24 h and 60–64% at 48 h. The most potent effect was observed with Hes100 + Gem10, yielding 38–42% at 24 h and 26–30% at 48 h (*p* < 0.001 vs. all single-agent groups), supporting a synergistic interaction.

### 3.2. Determination of IC_50_ Values and Evaluation of Drug Synergy via Chou–Talalay Method

**Figure 3:**
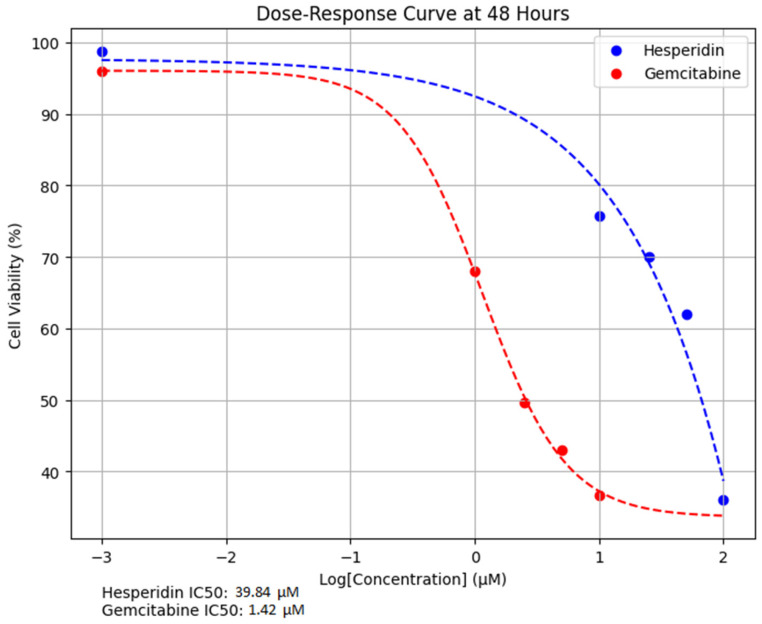
Dose–response curves of Hes and Gem in ISHIKAWA cells after 48 h of treatment. Data represent mean cell viability (%) ± SD from three independent experiments. IC_50_ values were estimated by nonlinear regression using a four-parameter logistic model, yielding 39.84 µM for Hes and 1.42 µM for Gem, confirming the greater potency of Gem at lower concentrations. For log-scale representation, zero values were replaced with 0.001 to enable transformation.

Dose–response curves demonstrated that both Hes and Gem induced time-dependent reductions in ISHIKAWA cell viability. The IC_50_ of Hes was calculated as 74.66 µM at 24 h and 39.84 µM at 48 h, whereas Gem displayed greater potency with IC_50_ values of 2.82 µM and 1.42 µM, respectively (Figure 3). These findings indicate that each compound exerts a dose- and time-dependent cytotoxic effect, with prolonged exposure enhancing drug activity.

To examine potential drug interactions, combination effects were initially evaluated using an additive model, where expected cell viability was derived as follows:

Expected viability (%) = (Hes viability × Gem viability)/100

For instance, in the Hes100 + Gem10 group, the expected viability was ≈13.20% [(36.00 × 36.67)/100], while the observed value was 28.00%. Because the experimental value exceeded the theoretical prediction, this outcome was interpreted as additive rather than synergistic. Comparable additive patterns were obtained for Hes10 + Gem1, Hes25 + Gem2.5, and Hes50 + Gem5.

**Figure 4:**
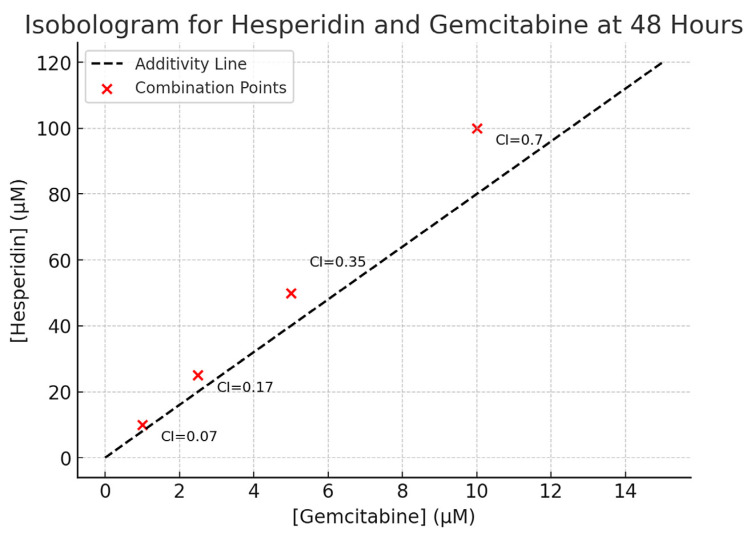
Isobologram illustrating the combinatorial effects of Hes and Gem on ISHIKAWA cells after 48 h. Red dots represent individual combination points with their respective Combination Index (CI) values calculated by the Chou–Talalay method. The dashed line denotes additivity. All combinations showed CI < 1, confirming synergistic interactions of varying strength: Hes10 + Gem1 (CI = 0.07), Hes25 + Gem2.5 (CI = 0.17), Hes50 + Gem5 (CI = 0.35), and Hes100 + Gem10 (CI = 0.70). Data were obtained from triplicate dose–response experiments and analyzed with CompuSyn software v1.0 (ComboSyn, Inc., Paramus, NJ, USA) using median-effect equation modeling.

When drug interactions were analyzed with the Chou–Talalay method, all tested combinations yielded Combination Index (CI) values < 1, indicating strong synergism (Figure 4). The discrepancy arises because the multiplicative model is based only on individual viability values at specific concentrations, while the Chou–Talalay approach integrates the entire dose–response curve, considering both IC_50_ and the slope parameter. This broader framework provides a more accurate characterization of pharmacological interactions. Thus, cases such as Hes100 + Gem10—where the observed viability exceeded the predicted additive value yet still produced CI < 1—highlight the greater reliability of the Chou–Talalay method for defining drug synergy.

### 3.3. Suppression of Angiogenesis via VEGF Inhibition

**Figure 5:**
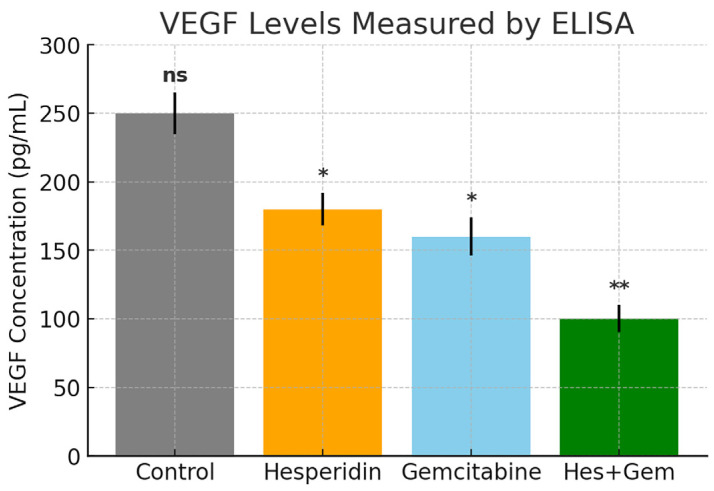
*VEGF* concentrations (pg/mL) in ISHIKAWA cell culture supernatants after 48 h treatments with Hes (100 µM), Gem (10 µM), and their combination, quantified by ELISA. The combination treatment (Hes + Gem) markedly reduced *VEGF* secretion compared to the control group (** *p* < 0.01), whereas Hes and Gem monotherapies resulted in moderate but statistically non-significant reductions (ns). Data are presented as mean ± SD from three independent experiments. Statistical comparisons were performed using one-way ANOVA followed by Tukey’s post hoc test (* *p* < 0.05, ** *p* < 0.01).

The anti-angiogenic effects of Hes and Gem were evaluated by quantifying *VEGF* secretion in cell culture supernatants using ELISA. Both Hes and Gem monotherapies significantly reduced *VEGF* levels compared with the control group (*p* < 0.05), demonstrating inherent anti-angiogenic activity. Importantly, the combination of Hes and Gem produced the most pronounced reduction in *VEGF* secretion, with a highly significant decrease relative to both the untreated control and the single-agent treatments (*p* < 0.01). These findings suggest that the combined treatment exerts a synergistic effect in suppressing angiogenesis, underscoring its potential to modulate pro-angiogenic signaling pathways (Figure 5).

### 3.4. Intracellular ROS Levels

Intracellular reactive oxygen species (ROS) levels in ISHIKAWA cells were quantified using the DCFH-DA fluorescence assay. Treatment with Hes (100 µM) significantly decreased ROS levels compared to the untreated control (** *p* < 0.01), demonstrating its strong antioxidant potential. In contrast, Gem (10 µM) markedly increased ROS production relative to control (* *p* < 0.05), consistent with its pro-oxidant and cytotoxic mechanism. Interestingly, co-treatment with Hes and Gem (Hes + Gem) induced a moderate ROS elevation; however, this increase was significantly lower than that observed with Gem alone (* *p* < 0.05), indicating that Hes partially attenuated Gem-induced oxidative stress.

**Figure 6:**
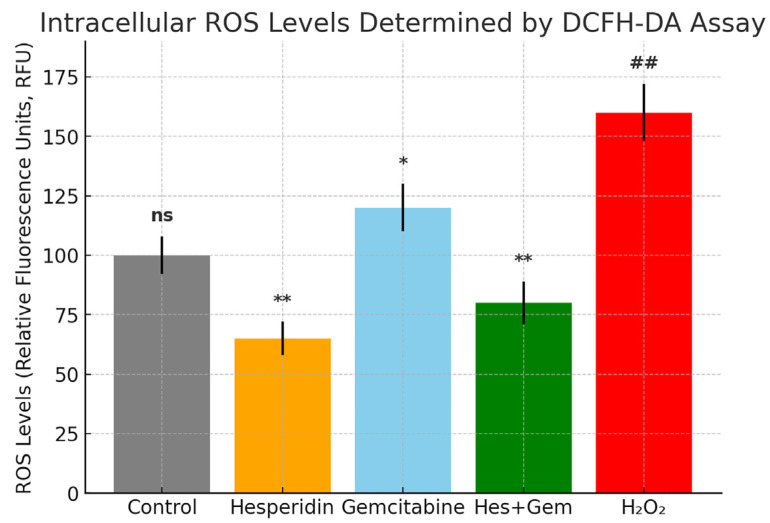
Intracellular ROS levels in ISHIKAWA cells were measured using the DCFH-DA fluorescence assay and expressed as relative fluorescence units (RFU). Treatment with Hes significantly reduced ROS levels compared to the control (** *p* < 0.01), confirming its antioxidant effect. In contrast, Gem treatment markedly increased ROS production relative to the control (* *p* < 0.05), indicating pro-oxidant activity. The combination treatment (Hes + Gem) resulted in a moderate elevation of ROS levels; however, these levels remained significantly lower than those observed with Gem alone (** *p* < 0.01). The positive control (H_2_O_2_, 100 µM) group exhibited the highest ROS levels (## *p* < 0.001 vs. all other groups). Data are presented as mean ± SD from three independent experiments. Statistical comparisons were performed using one-way ANOVA followed by Tukey’s post hoc test. ns, not significant.

All values are expressed as mean ± SD from three independent experiments performed in triplicate. Statistical comparisons were carried out using one-way ANOVA followed by Tukey’s post hoc test. These results collectively highlight the ROS-modulating role of Hes and its potential to counteract chemotherapy-induced oxidative stress (Figure 6).

### 3.5. Quantitative Luminometric Assessment of Caspase-3/7 Activity

**Figure 7:**
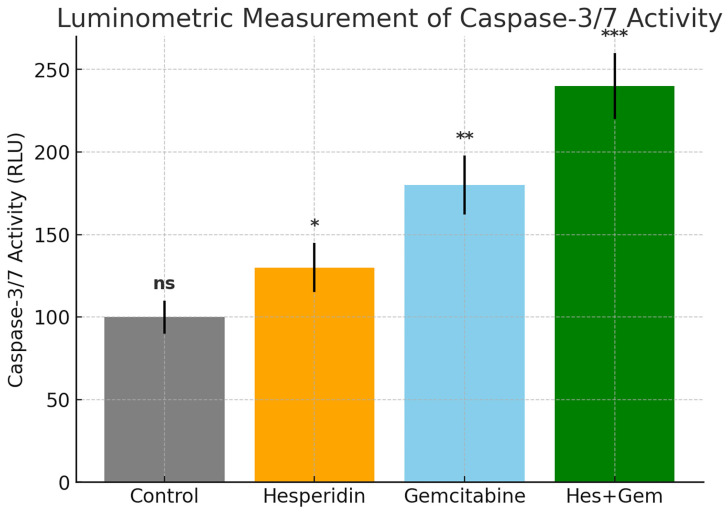
Quantitative luminometric measurement of *Caspase-3/7* activity in ISHIKAWA cells after 48 h treatment with Hes (100 µM), Gem (10 µM), and their combination. Caspase activity is expressed in relative light units (RLU) as mean ± standard deviation from three independent experiments. Hes treatment produced a modest but significant increase compared to control (* *p* < 0.05), whereas Gem treatment led to a stronger elevation (** *p* < 0.01). The combination group exhibited the highest caspase activation, showing a highly significant increase relative to both control and single-agent treatments (*** *p* < 0.001). Statistical analysis was performed using one-way ANOVA with Tukey’s post hoc test. ns, not significant.

*Caspase-3* and *Caspase-7* enzymatic activities in ISHIKAWA endometrial cancer cells were quantified using a luminometric assay following treatment. Gem monotherapy significantly increased *Caspase-3/7* activity compared to the control group (*p* < 0.01), while Hes alone produced a modest but statistically significant increase (*p* < 0.05). Notably, the combination of Hes and Gem induced the highest level of caspase-3/7 activation, showing a highly significant elevation relative to both the control and single-agent treatments (*p* < 0.001). These results demonstrate that the combined treatment exerts a strong pro-apoptotic effect, supporting a synergistic activation of apoptotic pathways (Figure 7).

### 3.6. Nuclei Using NucBlue Staining Findings

**Figure 8:**
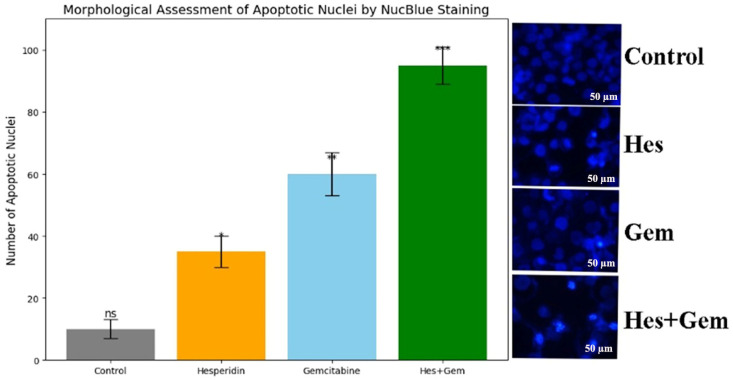
Quantitative analysis of apoptotic nuclei in ISHIKAWA cells stained with NucBlue following 48 h treatments. Nuclear morphological changes indicative of apoptosis, including chromatin condensation and fragmentation, were visualized under fluorescence microscopy at 20× magnification. Scale bars = 50 µm. Treatment with Hes and Gem significantly increased the number of apoptotic nuclei compared to the control (* *p* < 0.05 and ** *p* < 0.01, respectively), whereas the combination treatment induced the highest apoptotic index with a highly significant increase (*** *p* < 0.001). Data are presented as mean ± standard deviation from three independent experiments. Statistical significance was determined using one-way ANOVA followed by Tukey’s post hoc test. ns, not significant.

Nuclear morphological alterations associated with apoptosis were assessed in ISHIKAWA cells using NucBlue^®^ staining and fluorescence microscopy. Following treatment, hallmark apoptotic features such as chromatin condensation, nuclear fragmentation, and apoptotic body formation were clearly observed, with the most pronounced changes evident in the Hes + Gem combination group. Quantitative analysis demonstrated that both Hes and Gem monotherapies significantly increased the number of apoptotic nuclei compared to the control, while the combination treatment resulted in the highest apoptotic index (*p* < 0.001). These results confirm that the combined treatment exerts a potent pro-apoptotic effect at the morphological level (Figure 8).

### 3.7. Analysis of Gene Expression Levels by RT-qPCR

**Figure 9:**
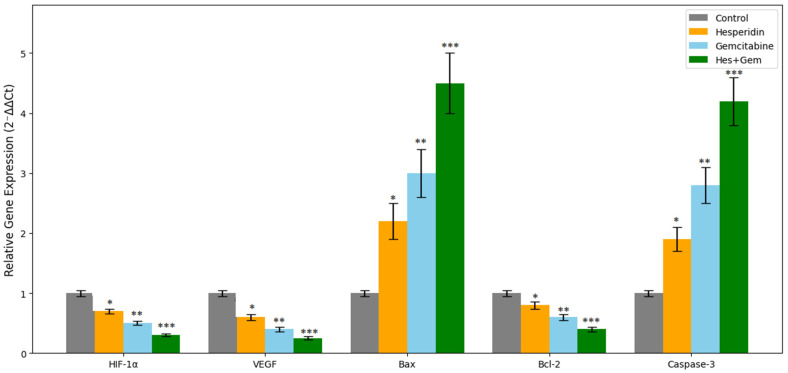
Relative mRNA expression levels of *HIF-1α*, *VEGF*, *Bax*, *Bcl-2*, and *Caspase-3* in ISHIKAWA cells after 48 h treatments with Hes (100 µM), Gem (10 µM), and their combination. Expression levels were quantified by *RT-qPCR* using the 2^−^ΔΔCt method. Both Hes and Gem treatments significantly downregulated the hypoxia- and angiogenesis-related genes *HIF-1α* and *VEGF*, with the combination group showing the most pronounced suppression (* *p* < 0.05, ** *p* < 0.01, *** *p* < 0.001). Conversely, the pro-apoptotic genes *Bax* and *Caspase-3* were significantly upregulated, particularly in the Gem and combination groups. Anti-apoptotic *Bcl-2* expression was consistently decreased across all treatment groups, with the strongest reduction observed in the combination group. Data are presented as mean ± SD from three independent experiments. Statistical significance was determined by one-way ANOVA followed by Tukey’s post hoc test.

The mRNA expression levels of hypoxia- and angiogenesis-related genes (*HIF-1α* and *VEGF*), as well as apoptosis-related genes (*Bax*, *Bcl-2*, and *Caspase-3*), were quantified using *RT-qPCR*. Both Hes and Gem treatments significantly downregulated *HIF-1α* and *VEGF* expression compared to the control, with the combination treatment producing the most pronounced suppression (*p* < 0.01). Conversely, the pro-apoptotic genes *Bax* and *Caspase-3* were markedly upregulated, particularly in the Gem and combination groups, with highly significant increases observed (*p* < 0.001). In parallel, the anti-apoptotic gene *Bcl-2* was significantly downregulated across all treatment groups, with the strongest reduction detected in the combination group (*p* < 0.01). Collectively, these findings demonstrate that the treatments effectively inhibit hypoxia- and angiogenesis-associated pathways while simultaneously promoting apoptosis in ISHIKAWA cells (Figure 9). For reference, baseline expression levels (mean ± SD) in untreated control cells were as follows: *Bax*: 1.00 ± 0.05, *Bcl-2*: 1.00 ± 0.04, *Caspase-3*: 1.00 ± 0.06, *HIF-1α*: 1.00 ± 0.03, and *VEGF*: 1.00 ± 0.05 (normalized to GAPDH). All fold changes reported for treated groups were calculated relative to these baseline values using the 2^−ΔΔCt^ method.

### 3.8. GO and Pathway Enrichment Analysis Findings

GO analysis of *HIF-1α*, *VEGF*, *Bax*, *Bcl-2*, and *Caspase-3* expression profiles in ISHIKAWA cells revealed significant enrichment across multiple biological categories, underscoring their functional roles in treatment responses.

- **BP (Biological Process):** The genes were mainly enriched in cellular response to hypoxia (GO: 0001666), angiogenesis (GO: 0001525), regulation of apoptosis (GO: 0042981), and programmed cell death (GO: 0012501). These enrichments support the observed modulation of hypoxic stress, angiogenesis, and apoptosis following treatment.
- **MF (Molecular Function):** The gene products were strongly associated with protein binding (GO: 0005515), transcription factor activity (GO: 0003700), and caspase activity (GO: 0004197). *Bax* and *Caspase-3* showed central roles in enzymatic activities driving apoptotic pathways.
- **CC (Cellular Component):** The proteins were predominantly localized in the mitochondria (GO: 0005739), the nucleus (GO: 0005634), and the cytoplasm (GO: 0005737). *Bax* and *Bcl-2* are key regulators at the mitochondrial membrane, whereas *HIF-1α* and *VEGF* function primarily in nuclear and extracellular compartments, respectively.

**Figure 10:**
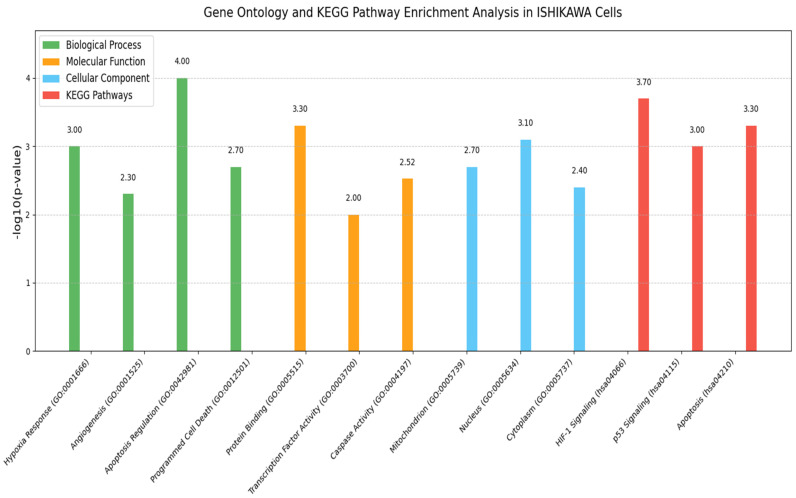
GO analysis revealed significant enrichment in biological processes such as cellular response to hypoxia (GO: 0001666), angiogenesis (GO: 0001525), and regulation of apoptosis (GO: 0042981). Molecular functions including protein binding (GO: 0005515) and caspase activity (GO: 0004197), as well as cellular components such as mitochondria (GO: 0005739) and nucleus (GO: 0005634), were also notably enriched. KEGG pathway analysis highlighted critical cancer-related signaling pathways, including HIF-1 signaling (hsa04066), p53 signaling (hsa04115), and apoptosis (hsa04210). Enrichment significance is presented as −log_10_ (*p*-value).

Complementary KEGG pathway enrichment analysis confirmed the involvement of these genes in major cancer-related pathways, including the *HIF-1* signaling pathway (hsa04066), the p53 signaling pathway (hsa04115), and the apoptosis pathway (hsa04210) (Figure 10).

## 4. Discussion

Endometrial cancer continues to pose a clinical burden due to its increasing prevalence and poor response to standard chemotherapy in advanced cases. In the present study, we evaluated the antiproliferative, antiangiogenic, oxidative stress-related, and pro-apoptotic properties of Hes and Gem, applied individually and in combination, in ISHIKAWA endometrial cancer cells. Our in vitro results demonstrated that while both agents exhibited significant antitumor effects, their combination produced superior outcomes through synergistic activity. The experimental design, which included cell viability (MTT assay), *VEGF* and ROS quantification, *Caspase-3/7* activation, nuclear morphology, and *RT-qPCR*-based gene expression analysis, allowed a mechanistic understanding of how these compounds regulate angiogenesis, apoptosis, and redox balance. Collectively, these findings highlight the potential of combining natural and synthetic agents to enhance therapeutic efficacy.

Both Hes and Gem suppressed proliferation, angiogenesis, and oxidative stress and promoted apoptosis in ISHIKAWA cells. Hes displayed dose-dependent cytotoxicity with defined IC_50_ values at 24 and 48 h, consistent with previous work showing that flavonoids inhibit proliferation by interfering with the cell cycle and inducing apoptosis [11,12]. Gem’s cytotoxicity is well-documented to result from DNA synthesis inhibition [13,14]. Importantly, their combined use resulted in a marked reduction in cell viability, with Chou–Talalay analysis confirming strong synergy [15]. This suggests that simultaneous disruption of multiple signaling pathways is more effective than single-agent therapy.

Our findings align with Cincin et al. [8], who demonstrated that Hes induces apoptosis in endometrial cancer cells via mitochondrial membrane potential disruption, *Caspase-3* activation, and *Bcl-2/Bax* modulation. Comparable synergistic effects of natural compounds with chemotherapeutics have been reported in other cancers. For instance, hispidin enhanced gemcitabine sensitivity in pancreatic cancer stem cells by reducing stemness and improving therapeutic response [16], while co-treatment with hesperidin and diosmin potentiated cisplatin-induced apoptosis in hepatocellular carcinoma cells without substantial toxicity to normal cells [17]. In endometrial cancer, hesperidin has been shown to suppress non-genomic estrogen receptor signaling, increase the *Bax/Bcl-2* ratio, and enhance *Caspase-3* activity [8]. To our knowledge, this is the first study to investigate the combined effects of hesperidin and gemcitabine in this cancer model, providing original evidence that simultaneous targeting of hypoxia, angiogenesis, and mitochondrial apoptosis pathways can enhance therapeutic outcomes.

The observed suppression of *VEGF* further confirms the anti-angiogenic potential of both Hes and Gem. Hes-mediated downregulation of *HIF-1α* and *VEGF* indicates interference with hypoxia-adaptive signaling, in agreement with earlier studies [18,19]. Similarly, Rahmani et al. emphasized the anti-angiogenic, pro-apoptotic, and anti-inflammatory roles of Hes via *ERK/MAPK* and *STAT3* inhibition [20], consistent with our data. Gem also suppressed angiogenesis, in line with prior studies reporting reduced tumor vascularization by chemotherapeutics [21,22]. Interestingly, contrary findings exist; Khan et al. reported Gem-induced *IL-8* secretion promoting angiogenesis in pancreatic cancer [23]. These discrepancies may reflect tumor type differences (endometrial vs. pancreatic), microenvironmental variations, and differences in Gem dosing or treatment duration. Furthermore, in vitro and in vivo settings may produce divergent outcomes due to distinct redox and growth factor conditions.

ROS assessment revealed that Hes functions as an antioxidant, whereas Gem enhances intracellular oxidative stress. Their combined treatment established a balanced ROS state, promoting apoptosis and inhibiting angiogenesis without excessive cytotoxicity. Gem-induced ROS elevation likely triggered mitochondrial depolarization, cytochrome c release, and caspase activation, while the antioxidant effects of Hes preserved redox homeostasis, preventing necrotic cell death but supporting programmed apoptosis. This dual regulation of ROS may be a central mechanism underlying the synergistic pro-apoptotic and anti-angiogenic effects of the Hes–Gem combination. Similar antioxidant-mediated modulation of chemotherapeutic side effects by flavonoids has been described in the literature [24,25,26].

Further confirmation of apoptosis induction was obtained through increased *Caspase-3/7* activity and nuclear changes observed with NucBlue staining. In parallel, *RT-qPCR* data revealed increased *Bax* and *Caspase-3* expression with concurrent *Bcl-2* downregulation, consistent with activation of the mitochondrial apoptotic pathway. These observations mirror those reported in gastric cancer cells treated with Hes [20]. Moreover, the enhancement of apoptosis with combination therapy is in line with previous evidence that flavonoids sensitize tumor cells to chemotherapy [27,28,29].

Overall, this study underscores the advantage of combining Hes and Gem, given their complementary cytotoxic and anti-angiogenic activities, alongside modulation of oxidative stress and apoptosis pathways. The major strength of this work lies in the integrated evaluation of proliferation, angiogenesis, oxidative stress, and apoptosis in a single model. Nonetheless, an important limitation is the absence of in vivo validation, which restricts direct translational interpretation. Future research should focus on validating these findings in animal models, exploring pharmacodynamic and bioavailability aspects of Hes, assessing drug–drug interactions, and delineating the involvement of key signaling pathways such as *ERK/MAPK*, *STAT3*, and estrogen receptor cascades to refine rational combination strategies.

## 5. Conclusions

Our findings indicate that Hes and Gem significantly influence ISHIKAWA endometrial cancer cells by suppressing cell growth, triggering apoptosis, inhibiting angiogenesis, and modulating oxidative stress responses. The combined application of these agents produced a pronounced synergistic cytotoxic effect, reflected in elevated caspase activation, upregulation of Bax, downregulation of Bcl-2, and reduced expression of VEGF and HIF-1α. Hes contributed not only to pro-apoptotic activity but also to antioxidant defense, suggesting a dual role in enhancing chemotherapy efficacy while potentially alleviating oxidative damage. Overall, the data support the potential of Hes as an adjuvant agent capable of reinforcing conventional chemotherapeutic regimens. Further preclinical animal experiments and clinical investigations are warranted to clarify pharmacokinetic properties, safety, and the translational feasibility of this combined therapeutic approach for endometrial cancer.

## Figures and Tables

**Table 1:** Primer sequences used for RT-qPCR.

Gene	Forward (5′–3′)	Reverse (5′–3′)
*HIF-1α*	*GAACGTCGAAAAGAAAAGTCTC*	*CCTTATCAAGATGCGAACTC*
*VEGF*	*AGGGCAGAATCATCACGAAGT*	*AGGGTCTCGATTGGATGGC*
*Bax*	*TGGAGCTGCAGAGGATGATTG*	*GACTCGCTCAGCTTCTTGGT*
*Bcl-2*	*GGTGGGGTCATGTGTGTGG*	*CGGTTCAGGTACTCAGTCATCC*
*Caspase-3*	*TGGAGCGAATCAATGGACTCT*	*TCCCACTGTCTGTCTCAATG*
*GAPDH*	*ACCACAGTCCATGCCATCAC*	*TCCACCACCCTGTTGCTGTA*

**Table 2:** Genes included in GO and KEGG enrichment analyses.

Gene Symbol	Full Name	Functional Role
*HIF1A*	*Hypoxia-inducible factor 1-alpha*	*Transcription factor regulating hypoxia response*
*VEGFA*	*Vascular endothelial growth factor A*	*Key mediator of angiogenesis*
*BAX*	*BCL2-associated X protein*	*Pro-apoptotic regulator in mitochondria*
*BCL2*	*B-cell lymphoma 2*	*Anti-apoptotic regulator*
*CASP3*	*Caspase-3*	*Executioner caspase in apoptosis*

## Data Availability

All details about the study can be obtained from the corresponding author. The raw data for ROS, caspase activity, and gene expression analyses are available from the corresponding author upon reasonable request.
